# Development of Electrospun Poly(3-hydroxybutyrate-*co*-3-hydroxyvalerate) Monolayers Containing Eugenol and Their Application in Multilayer Antimicrobial Food Packaging

**DOI:** 10.3389/fnut.2020.00140

**Published:** 2020-09-03

**Authors:** Kelly J. Figueroa-Lopez, Luis Cabedo, Jose M. Lagaron, Sergio Torres-Giner

**Affiliations:** ^1^Novel Materials and Nanotechnology Group, Institute of Agrochemistry and Food Technology (IATA), Spanish National Research Council (CSIC), Paterna, Spain; ^2^Polymers and Advanced Materials Group (PIMA), Universitat Jaume I (UJI), Castellón de la Plana, Spain

**Keywords:** PHA, essential oils, multilayer, antimicrobial activity, electrospinning

## Abstract

In this research, different contents of eugenol in the 2.5–25 wt.% range were first incorporated into ultrathin fibers of poly(3-hydroxybutyrate-*co*-3-hydroxyvalerate) (PHBV) by electrospinning and then subjected to annealing to obtain antimicrobial monolayers. The most optimal concentration of eugenol in the PHBV monolayer was 15 wt.% since it showed high electrospinnability and thermal stability and also yielded the highest bacterial reduction against *Staphylococcus aureus* (*S. aureus*) and *Escherichia coli* (*E. coli*). This eugenol-containing monolayer was then selected to be applied as an interlayer between a structural layer made of a cast-extruded poly(3-hydroxybutyrate) (PHB) sheet and a commercial PHBV film as the food contact layer. The whole system was, thereafter, annealed at 160°C for 10 s to develop a novel multilayer active packaging material. The resultant multilayer showed high hydrophobicity, strong adhesion and mechanical resistance, and improved barrier properties against water vapor and limonene vapors. The antimicrobial activity of the multilayer structure was also evaluated in both open and closed systems for up to 15 days, showing significant reductions (R ≥ 1 and < 3) for the two strains of food-borne bacteria. Higher inhibition values were particularly attained against *S. aureus* due to the higher activity of eugenol against the cell membrane of Gram positive (G+) bacteria. The multilayer also provided the highest antimicrobial activity for the closed system, which better resembles the actual packaging and it was related to the headspace accumulation of the volatile compounds. Hence, the here-developed multilayer fully based on polyhydroxyalkanoates (PHAs) shows a great deal of potential for antimicrobial packaging applications using biodegradable materials to increase both quality and safety of food products.

## Introduction

Active antimicrobial packaging is one of the most relevant emerging technologies in the food industry. It aims to control enzymatic, chemical, physical, and microbiological reactions that deteriorate food through the inclusion of active substances and their controlled release to the surface in contact with food (1, 2). Biodegradable polymers have recently emerged as an alternative to gradually replace the use of petrochemical polymers in packaging applications, which are known to generate irreversible environmental damage (3, 4). Furthermore, polymers used in packaging can also be obtained from the valorization of food-processing by-products and agricultural or industrial wastes, being more sustainable and cost-effective (5).

Polyhydroxyalkanoates (PHAs) are among the most commonly used thermoplastic biopolyesters in food packaging applications because they have similar properties to some conventional non-degradable plastics (6, 7). PHAs can be obtained from over 155 monomer subunits through fermentation by some bacteria from different renewable carbon sources that are accumulated as intracellular storage granules (8). For instance, Bhatia et al. (9) obtained high biomass (Y_x/s_, 0.31 g/g) and PHA (Y_p/s_, 0.14 g/g) yields using *Ralstonia eutropha* 5,119 bacteria and *Miscanthus* biomass hydrolysate (MBH) as carbon source. In another research work, Bhatia et al. (10) obtained a high PHB production (1.24 g/L) with 2% (w/v) starch as carbon source, using a *Escherichia coli* (*E. coli*) strain produced using different plasmids containing the amylase gene of *Panibacillus* sp. and PHB synthesis genes from *Ralstonia eutropha*. Park et al. (11) studied different PHA-producing strains, concluding that *Halomonas* sp. YLGW01 produced the highest amount of PHB [94.6 ± 1.8% (w/w)] using fructose as carbon source. Hong et al. (12) determined the optimal growth and production conditions, environments with different salinity, carbon sources, and nitrogen sources of the *Vibrio proteolyticus* strain in the PHA production. It was concluded that the use of a medium containing 2% (w/v) fructose, 0.3% (w/v) yeast extract, and 5% (w/v) sodium chloride (NaCl) in M9 minimal medium resulted in high PHA (54.7%) and biomass (4.94 g/L) contents. The structure and physical properties of PHAs vary depending on the bacteria specie, substrate (carbon source), and cultivation conditions (13). PHAs are a typically linear aliphatic polyesters consisting of repetitive hydroxy acids (HAs) connected together by an ester bond (14). According to the number of carbon atoms in the monomers, PHAs are classified in groups of short chain length (*scl*-PHAs), which consists of 3–5 carbon atoms (C3–C5), and medium chain length (*mcl*-PHAs) with 6–14 carbon atoms (C6–C14) (15, 16). Microbial synthesis of PHAs have the capacity to produce copolymers from mixed substrates by different methods, such as metabolic engineering, co-culture of microbes, and feeding of the various precursors during fermentation (17–19). Typical examples of *scl*-PHAs are poly(3-hydroxybutyrate) (PHB), poly(3-hydroxybutyrate-*co*-3-hydroxyvalerate (PHBV), and poly(3-hydroxybutyrate-*co*-4-hydroxybutyrate) [P(3HB-*co*-4HB)]. The most representative *mcl*-PHAs are poly(3-hydroxybutyrate-*co*-3-hydroxyhexanoate) [P(3HB-*co*-3HHx)] and poly(3-hydroxyhexanoate-*co*-3-hydroxyoctanoate) [P(3HHx-*co*-3HO)] (20, 21). PHB is the most abundant type of PHA but it is brittle and shows low thermal stability, which limits their industrial applications. For this reason, PHB is currently being replaced by PHBV due to the better processability and physical properties of the copolyester, such as higher flexibility improved toughness, and lower melting point and level of crystallinity (22, 23). PHAs can be processed by different melt-processing techniques such as injection molding (24), extrusion (25), and compression molding (26). Electrospinning is one of the latest technologies to process PHAs in the form of monolayer and multilayer films by the application of high electric voltages and annealing treatments, having the main advantage to operate at room temperatures (27, 28). The latter process, therefore, opens up the incorporation thermolabile substances into electrospun biopolymer films including natural extracts (29).

Essential oils are natural substances obtained by secondary metabolite of plants such as flowers, stems, leaves, seeds, etc. (30). Different studies have reported multiple active and bioactive properties for essential oils, including antibacterial, antimitotic, antisepticise, and antiviral properties, due to the presence of aldehydes and phenols, for instance carvacrol, eugenol, and thymol (31). Among them, eugenol (C_10_H_12_O_2_) is the main active phenolic compound present in the clove essential oil (32), which shows strong antibacterial activity against a wide range of Gram negative (G-) bacteria, including *E. coli, Salmonella typhimurium, Pseudomonas aeruginosa*, and also Gram positive (G+) bacteria, including *Staphylococcus aureus* (*S. aureus*) and *Listeria monocytogenes* (*L. monocytogenes*) (33, 34). The action of essential oils against bacteria is based on interfering chemically with the synthesis or function of principal components of bacteria as well as blocking their antimicrobial resistance mechanisms. In this way, these natural antimicrobial substances are known to affect the bacterial protein biosynthesis, deoxyribonucleic acid (DNA) replication and repair, destroy cell membrane and wall or inhibit metabolism and structural integrity (35–38). However, due to its high volatility, water insolubility, poor oxidation, and high thermal sensitivity, essential oils habitually need to be protected to preserve their activity (39). In this way, the electrospinning technology offers multiples advantages for the nanoencapsulation of essential oils such as the use of mild temperatures (40) and materials with high surface-to-volume ratios and controlled porosity (41, 42).

Multilayer films are combinations of different layers typically based on materials with dissimilar properties glued together to fulfill functions that monomaterials do not offer (43, 44). Multilayers are often composed of “structural” and “barrier” layers, usually on the outside and the inside, respectively. An “active” layer can additionally be added either on the outside or inside, depending on the application needs. Layers of adhesive polymers, also named “tie layers”, are used as glue between the different layers where necessary. The purpose of using multilayer systems is to improve the properties of the food packaging due to the majority of polymers, particularly biopolymers, and paper present poor water resistance, low oxygen barrier, and reduced mechanical performance (45–47). As a result, the multilayer present higher advantages than monolayer systems in terms of industrial process and applications in food packaging (48, 49). In this context, the electrospinning technology can result very effective to prepare active and bioactive multilayer structures with sustained released capacity by means of coatings or interlayers containing entrapped substances with functionality (50). For instance, electrospun zein fibers in the form of coatings (51) or interlayers (52) have significantly improved the gas barrier properties and provided active performance to polylactide (PLA) films. Similarly, Fabra et al. (53, 54) followed the same strategy to develop multilayer structures based on PHAs biopolyesters and electrospun zein nanofibers. Cherpinski et al. improved the water resistance of paper (55) and nanopaper (56) by coatings of electrospun films of PHB and other biopolymers. Quiles-Carrillo et al. (57) produced bioactive films with sustained antioxidant release capacity by means of multilayer structures of PLA containing electrospun fibers with gallic acid (GA). More recently, Akinalan Balik et al. (58) developed high-barrier multilayer films using an electrospun pectin-based film applied as an interlayer between two external layers of PHBV.

The main objective of this study was to develop a novel multilayer system based on PHBV with antimicrobial properties by electrospinning. To achieve this end, electrospun mats of PHBV fibers containing different amounts of eugenol were characterized and the most efficient was selected to coat PHB sheets. Thereafter, a food contact layer of PHBV was put on the eugenol-containing electrospun layer and the whole structure was subjected to annealing at mild temperature to form a multilayer without the need to use a tie layer. The morphological, thermal, mechanical adhesion, and barrier properties to water and limonene vapors were determined. Finally, the antimicrobial activity of the multilayers against *S. aureus* and *E. coli* in both an open and closed system was tested for 15 days in order to ascertain their potential in active food packaging.

## Materials and Methods

### Materials

Bacterial aliphatic copolyester PHBV was ENMAT^TM^ Y1000P, produced by Tianan Biologic Materials (Ningbo, China) and supplied by Ocenic Resins SL (Valencia, Spain). The biopolymer resin was delivered in pellets with a true density of 1.23 g/cm3. The molar fraction of HV in the copolymer is 2–3%, while the molecular weight (M_W_) is ~2.8 × 10^5^ g/mol. Bacterial aliphatic homopolyester PHB was provided in pellets by Biomer (Krailling, Germany) as P226F. It has a density of 1.25 g/cm^3^, a melt flow rate (MFR) of 10 g/10 min at 180°C and 5 kg, a M_W_ of 500 kDa, and a polydispersity index (PI) of 2. A 25-μm film of PHBV with a molar fraction of HV of 8 mol.% was purchased at GoodFellow Cambridge Limited (Huntindgon, UK) with the commercial reference BV301025. All the PHA grades are certified by the manufacturers both as compostable and food contact. 2,2,2-trifluorethanol (TFE), with 99% purity, and eugenol, ReagentPlus®, with 99% purity, were both purchased from Sigma-Aldrich S.A. (Madrid, Spain). Glycerol, phosphate buffered saline (PBS), and tryptic soy broth (TSB) were provided by Conda Laboratories (Madrid, Spain).

### Preparation of the Structural Layer by Cast Extrusion

The PHB pellets were cast-extruded into sheets using a cast-roll machine MINI CAST 25 from EUR.EX.MA (Venegono, Italy). The extrusion speed was set at 25 rpm and the temperature profile, from the feeding zone to die head, was adjusted to 180–175–170–170–165–165–160°C. PHBV sheets with an average thickness of ~500 μm were obtained by adjusting the speed of the calendar and the drag.

### Preparation of the Multilayers by Electrospinning and Annealing

#### Solutions for Electrospinning

Different solutions for electrospinning were prepared by dissolving 10% in weight (wt.%) of PHBV in TFE at room temperature. Eugenol was incorporated into the solutions at 2.5, 5, 10, 15, 20, and 25 wt.% in relation to the PHBV content.

#### Electrospinning

The PHBV solutions containing eugenol were processed in a Fluidnatek® LE10 electrospinning benchtop equipment from Bioinicia S.L. (Valencia, Spain) with a variable high-voltage 0–30 kV power supply and a motorized scanning injector. The PHBV solutions were transferred to a 30-mL plastic syringe and coupled by a Teflon tube to a stainless-steel needle (Ø = 0.9 mm) that was connected to the power supply. Thus, each PHBV solution was electrospun at room temperature, that is, 25°C, for 2 h under a steady flow-rate of 6 mL/h, scanning horizontally toward the cast-extruded PHB sheet attached to the metallic collector. The applied voltage was 15 kV while the distance between the injector and collector was optimal at 15 cm. An electrospun mat of PHBV without eugenol was also deposited on the PHB sheets in the same conditions to produce the control multilayer. The same process was also carried out to collect individual interlayers on the collector without using the PHB sheets. In all cases, the resultant electrospun materials were stored in a desiccator at 25°C and 0% relative humidity (RH) for, at least, 48 h.

#### Annealing

**Figure 1:**
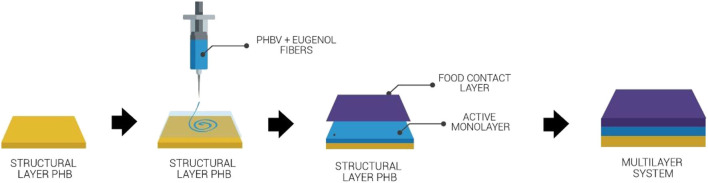
Process to obtain the multilayer system of poly(3-hydroxybutyrate) (PHB) sheet/electrospun poly(3-hydroxybutyrate-*co*-3-hydroxyvalerate) (PHBV) interlayer containing eugenol/PHBV film.

The multilayer systems were produced according to the process schemed in Figure 1. Briefly, once the electrospun PHBV mats containing eugenol were coated on the cast-extruded PHB sheets, the food contact film of PHBV was deposited on the electrospun layer and the whole system was subjected to annealing. This thermal post-treatment was performed at 160°C, below the biopolymer's melting point, for 10 ± 1 s, without pressure, using a hydraulic press 4122-model from Carver, Inc. (Wabash, IN, USA). The resultant multilayers were finally air cooled at room temperature. These conditions were selected based on our previous studies for thermally post-processing PHAs (59). The same process was applied to single electrospun monolayers of PHBV for comparison purposes.

### Characterization of the Electrospun Monolayers and Multilayer

#### Thickness

Before testing, the thicknesses of all the monolayer and multilayer structures were measured using a digital micrometer (S00014, Mitutoyo, Corp., Kawasaki, Japan) with ±0.001 mm accuracy. Measurements were performed and averaged in five different points with two in each end and one in the middle.

#### Morphology

The morphology of the electrospun fibers and the cross-sections of the films were examined by scanning electron microscopy (SEM). Cryofracture of the films was previously carried out by immersing the samples in liquid nitrogen. The SEM micrographs were obtained using a Hitachi S-4800 electron microscope (Tokyo, Japan) at an accelerating voltage of 10 kV and a working distance of 8–10 mm. Prior to examination, all the samples were fixed to beveled holders using a conductive double-sided adhesive tape and sputtered for 3 min with a mixture of gold–palladium mixture under vacuum. Fiber sizes and film thickness were determined by means of the ImageJ 1.50i software using the SEM micrographs in their original magnification. At least 25 micrographs of each sample were used for the measurements.

#### Thermal Analysis

Thermogravimetric analysis (TGA) of the neat eugenol oil and PHBV films was performed under nitrogen atmosphere in a Thermobalance TG-STDA Mettler Toledo model TGA/STDA851e/LF/1600 analyzer (Greifensee, Switzerland). TGA curves were obtained after conditioning the samples in the sensor for 5 min at 30°C. The samples were then heated from 25 to 700°C at a heating rate of 10°C/min. All the thermal tests were carried out in triplicate.

#### Water Contact Angle

The wettability of the interlayers and multilayers surface was evaluated by the measurement of the dynamic water contact angle (WCA) in an Optical Tensiometer (Theta Lite, Staffordshire, UK). Five droplets were seeded at 5 μL/s on the surfaces of each studied sample sizing 2 × 5 cm^2^ and the WCA values were averaged. Measurements were performed at 25°C.

#### Mechanical Adhesion

**Figure 2:**
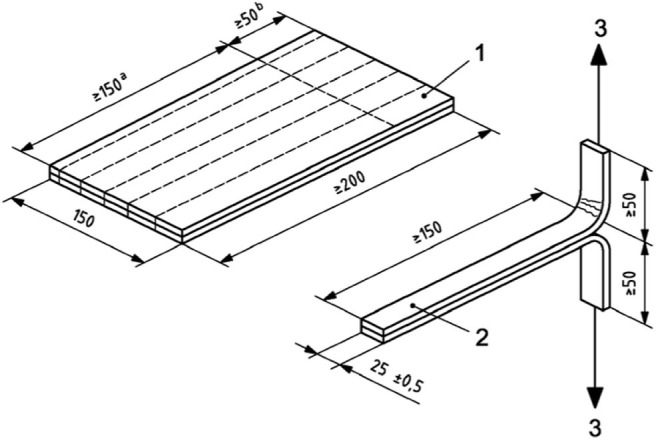
Sample geometry for the T-peel adhesion test.

The adhesion capacity of the electrospun PHBV layer was assessed by a T-peel test according to ISO 11339:2010. To this end, the multilayer structure was assembled by sandwiching an electrospun PHBV interlayer in between two cast-extruded PHB films (200 mm × 150 mm) according to the sample geometry shown in Figure 2. Prior to the annealing step, a 150 mm × 50 mm Teflon film was inserted along the edge of the PHB sheets in order to prevent adhesion in the grip zone of the specimens (see zone 1 in Figure 2). Adhesion tests consisted on measuring the force required to peel the two PHB sheets adhered by the electrospun PHBV interlayer by means of a uniaxial tensile test in a universal testing machine (Shimadzu AGS-X 500 N, Shimadzu Corporation, Kyoto, Japan) at room temperature with a cross-head speed of 10 mm/min.

#### Water Vapor Permeance

The water vapor permeability (WVP) of the different multilayers was determined according to the ASTM 2011 gravimetric method using Payne permeability cups (Elcometer SPRL, Hermelle/s, Liege, Belgium) of 3.5 cm diameter. One side of the films was exposed to 100% RH by avoiding direct contact with liquid water. Then, the cups containing the films were secured with silicon rings and stored in a desiccator at 25°C and 0% RH. The cups were weighed periodically after the steady state was reached. Water vapor permeation rate corresponded to the slope value of the steady state line of time vs. weight loss per unit area and the weight loss was calculated as the total loss minus the loss through the sealing. Water vapor permeance was obtained by correcting the water vapor permeation rate for the permeant partial pressure. Measurements were done in triplicate for each type of samples.

#### Limonene Vapor Permeance

Limonene permeability (LP) was measured as described above for WVP. For this, 5 mL of _D_-limonene was placed inside the Payne permeability cups and the cups containing the films were placed at controlled room conditions of 25°C and 40% RH. The limonene vapor permeation rates were estimated from the steady-state permeation slopes and the weight loss was calculated as the total cell loss minus the loss through the sealing. Limonene permeance was obtained by correcting the limonene vapor permeation rate for the permeant partial pressure. Tests were conducted in triplicate.

#### Antimicrobial Tests

The antimicrobial performance of the monolayers and multilayer was evaluated according to the Japanese Industrial Standard (JIS) Z 2801:2010. *S. aureus* CECT240 (ATCC 6538P) and *E. coli* CECT434 (ATCC 25922) strains were obtained from the Spanish Type Culture Collection (CECT) (Valencia, Spain) and stored in phosphate buffered saline (PBS) with 10 wt.% tryptic soy broth (TSB) and 10 wt.% glycerol at −80°C. Previous to each study, a loopful of bacteria was transferred to 10 ml of TSB and incubated at 37°C for 24 h. A 100-μL aliquot from the culture was again transferred to TSB and grown at 37°C to the mid-exponential phase of growth. An approximate count of 5 × 10^5^ colony forming units (CFU)/mL of a culture having an absorbance value of 0.20 as determined by optical density at 600 nm using an UV–Vis spectrophotometer VIS3000 (Dinko, Instruments, Barcelona, Spain) was used. A microorganism suspension of *S. aureus* and *E. coli* was applied onto the test monolayer and multilayer films sizing 1.5 cm × 1.5 cm containing eugenol that were placed in hermetically closed and open bottles, the here so-called closed and open systems, respectively. The electrospun PHBV monolayer without eugenol was used as the control film since it shows no antimicrobial activity (60, 61). After incubation at 24°C and, at least, 95% RH, for 24 h, bacteria were recovered with PBS, 10-fold serially diluted, and incubated at 37°C for 24 h in order to quantify the number of viable bacteria by conventional plate count. The antimicrobial activity was evaluated at 1 (right after the film production), 8, and 15 days in both the closed and open systems. The value of the antimicrobial reduction (*R*) was calculated using the expression:

(1) $$\begin{array}{l} R=\left[\log\left(\frac{B}{A}\right)-log\left(\frac{C}{A}\right)\right]=\log\left(\frac{B}{C}\right) \end{array}$$

Where A is the mean of bacterial counts of the control sample immediately after inoculation, B is the mean of bacterial counts of the control sample after 24 h, and C is the mean of bacterial counts of the test sample after 24 h. Antimicrobial activity was evaluated with the following assessment: Non-significant (*R* < 0.5), slight (*R* ≥ 0.5 and < 1), significant (R ≥ 1 and < 3), and strong (R ≥ 3) (62).

### Statistical Analysis

The thermal and mechanical properties, contact angle measurements, and water and limonene vapor permeance were evaluated by analysis of variance (ANOVA) and a multiple comparison test (Tukey) with 95% significance level (*p* ≤ 0.05). For this purpose, the software OriginPro8 (OriginLab Corporation, Northampton, MA, USA) was used.

## Results and Discussion

### Characterization and Selection of the Electrospun Active PHBV Monolayer

#### Morphology

**Figure 3:**
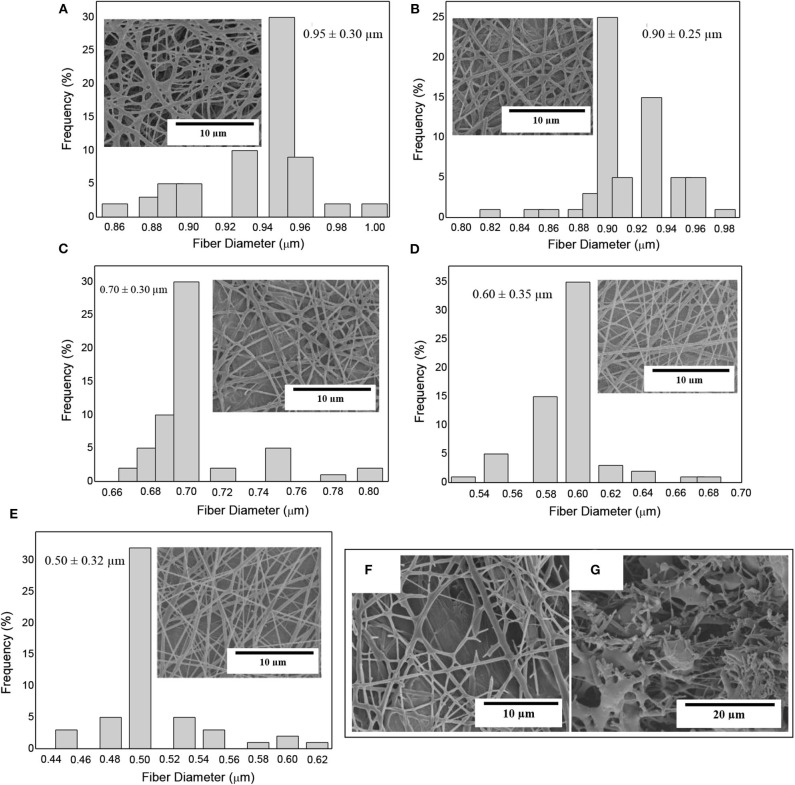
Scanning electron microscopy (SEM) micrographs of electrospun fiber mats of poly(3-hydroxybutyrate-*co*-3-hydroxyvalerate) (PHBV) containing eugenol and their histograms: **(A)** Neat PHBV; **(B)** 2.5 wt.%; **(C)** 5 wt.%; **(D)** 10 wt.%; **(E)** 15 wt.%; **(F)** 20 wt.%; **(G)** 25 wt.%.

Figure 3 shows the morphology and the fiber diameters histogram of the electrospun PHBV fiber mats containing eugenol prior to annealing. In Figure 3A one can observe that the neat PHBV fibers presented a mean diameter of ~0.95 μm. Figure 3B corresponds to electrospun PHBV fibers with 2.5 wt.% of eugenol, which had a mean diameter of ~0.90 μm. In Figure 3C it can be seen that the fibers containing 5 wt.% of eugenol showed a mean diameter of ~0.7 μm, whereas the diameters in the fibers containing 10 wt.% of eugenol was ~0.6 μm (Figure 3D) and in the fibers with 15 wt.% eugenol was ~0.5 μm (Figure 3E). As it can be seen in the histograms, the fiber size decreased as the eugenol concentration increased. This effect can be ascribed to the plasticizing effect and reduction in the solution viscosity due to the presence of the oily substance (63, 64). Indeed, solution viscosity plays a major role in the fiber diameter during electrospinning and solutions with lower viscosities tend to result in fibers with lower diameters (65, 66). One can also observe in the SEM micrographs that higher concentrations of eugenol impaired the formation of PHBV fibers during electrospinning due to the potential agglomeration of the essential oil. In particular, Figure 3F shows that 20 wt.% of eugenol resulted in a partial breakage of the PHBV fibers, while 25 wt% yielded fibers with beaded regions (see Figure 3G). Similar results were obtained during the electrospinning of PHBV with various essential oils, in which high contents of oil molecules prevented the formation of homogeneous fibers since the surface tension in the charged jet changed into droplets (59, 67). Therefore, eugenol contents of up to 15 wt.% led to electrospun PHBV fibers with smooth surfaces and free of defects or beaded regions so that higher contents of the essential oil were ruled out of the study.

**Figure 4:**
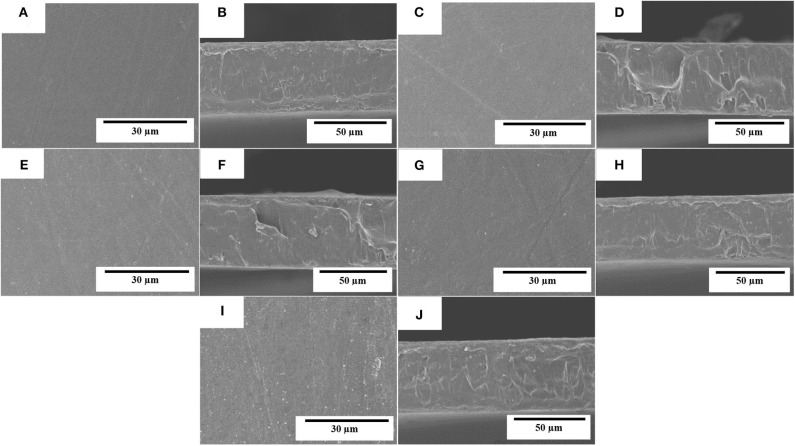
Scanning electron microscopy (SEM) micrographs of the electrospun monolayers of poly(3-hydroxybutyrate-*co*-3-hydroxyvalerate) (PHBV) containing eugenol: **(A,B)** neat PHBV; **(C,D)** 2.5 wt.%, eugenol; **(E,F)** 5 wt.%; **(G,H)** 10 wt.%; **(I,J)** 15 wt.% eugenol. Scale markers of 30 μm for top views and of 50 μm for cross-sections.

**Figure 5:**
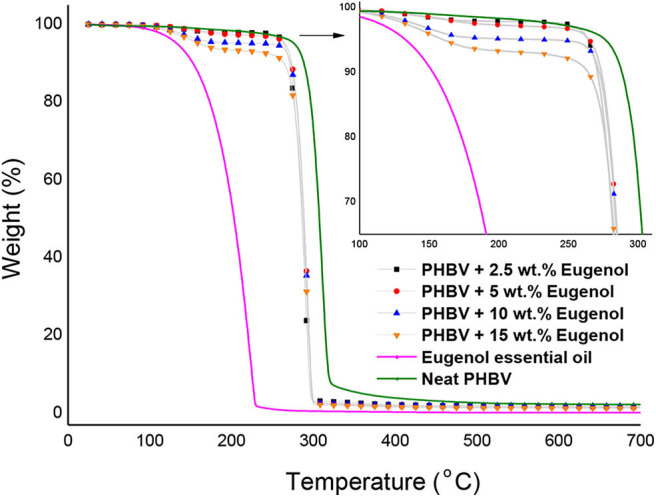
Thermogravimetric analysis (TGA) curves of eugenol and electrospun monolayers of poly(3-hydroxybutyrate-*co*-3-hydroxyvalerate) (PHBV) containing eugenol.

The electrospun fibers mats were turned into actual films by the application of annealing at 160°C for 10 s. The morphologies of the top views and cross-sections of the PHBV films containing eugenol were analyzed by SEM and they are gathered in Figure 4. It can be observed that the films of neat PHBV (Figures 4A,B) and PHBV containing 2.5 wt.% (Figures 4C,D), 5 wt.% (Figures 4E,F), 10 wt.% (Figures 4G,H), and 15 wt.% (Figures 4I,J) of eugenol were very similar. In all cases, the individual electrospun PHBV fibers successfully coalesced and merged without melting. All the films presented uniform and homogeneous surfaces, which is an indication of the good cohesion of the materials. The cross-sections of the films presented nearly the same thicknesses (~70 μm), demonstrating that the addition of eugenol did not affect the morphology of the films. Similar observations were reported by Shen and Kamdem (68) who developed active biodegradable films of chitosan containing 10–30% wt/wt of citronella essential oil and cedarwood oil by casting and solvent-evaporation methods, showing a uniform film thicknesses. On the other hand, however, Haghighi et al. (69) observed a changed in the microstructure of films based on chitosan and gelatin after the incorporation of the essential oils eugenol and ginger. The authors observed the presence of pores in the films that indicated the occurrence of collapsed oil droplets due to the separation of aqueous phase during the drying step as result of their hydrophobic nature. In this way, the annealed electrospun mats present the advantage to develop more homogenous and continuous films containing essential oils over the casting method due to the drying steps are not necessary.

#### Thermal Stability

Figure 5 shows the TGA curves obtained for the neat eugenol and the electrospun PHBV films containing eugenol. Table 1 gathers the most relevant thermal stability parameters obtained from the TGA curves, that is, the onset degradation temperature (T_onset_), measured at 5% of weight loss (T_5%_), the degradation temperature (T_deg_), and the mass loss at T_deg_. One can observe that neat eugenol initiated thermal decomposition at ~110°C, showing a T_5%_ of 121.3°C and presenting a maximum degradation peak, that is, T_deg_, at 203.5°C with a mass loss at T_deg_ of 86.68%. This result confirms that this essential oil is thermally unstable and it cannot be processed at high temperatures. In this regard, Shao et al. (70) reported a thermal value for the maximum degradation of eugenol at 195°C, which is characteristic of its low-M_W_ substances. It has been reported that eugenol decomposition is based on a first low-intense mass loss around 44°C associated to the release of volatiles molecules and the second mass loss occurs at 200°C where it takes places the complete degradation of the organic compounds (71). As opposite, one can observe that the electrospun PHBV films were relatively stable, showing values of T_5%_ of 276.6°C and T_deg_ of 304.7°C with a mass loss at T_deg_ of 61.01%. The films containing 2.5 wt.% and 5 wt.% of eugenol showed very similar thermal decomposition profiles, presenting their onset of degradation at 264.2 and 265°C, respectively, and exhibiting T_deg_ values of 291.4 and 293.3°C with respective mass losses of 71.18 and 72.14%. The electrospun PHBV film containing 10 wt.% of eugenol started thermal decomposition from 245.8°C, presenting a T_deg_ value of 293.1°C with a mass loss of 74.60%. Finally, in the case of the electrospun PHBV film containing 15 wt.% of eugenol, it showed a significantly lower value of T_5%_, that is, 160.8°C, while T_deg_ was similar to the other films, that is, 293.3°C (76.36%). The reduction observed in the onset of thermal degradation can be attributed to the low thermal stability of eugenol.

**Table 1:** Thermal properties of eugenol and electrospun monolayers of poly(3-hydroxybutyrate-*co*-3-hydroxyvalerate) (PHBV) containing eugenol in terms of: temperature at 5% weight loss (T_5%_), degradation temperature (T_deg_), mass loss at T_deg_, and mass loss at 230°C.

**Sample**	**T_**5%**_ (^**°**^C)**	**T_**deg**_ (^**°**^C)**	**Mass loss at T_**deg**_ (%)**	**Mass loss at 230^**°**^C (%)**
Eugenol	121.3 ± 1.5^a^	203.5 ± 1.1^a^	86.68 ± 0.7^a^	98.12 ± 1.0^a^
PHBV	276.6 ± 0.9^b^	304.7 ± 1.6^b^	61.01 ± 1.2^b^	2.13 ± 0.7^b^
PHBV + 2.5 wt.% Eugenol	264.2 ± 2.1^c^	291.4 ± 0.7^c^	71.18 ± 0.9^c^	2.15 ± 1.1^b^
PHBV + 5 wt.% Eugenol	265.0 ± 2.3^c^	293.3 ± 2.5^c^	72.14 ± 2.0^c^	2.88 ± 2.0^a^
PHBV + 10 wt.% Eugenol	245.8 ± 0.7^d^	293.1 ± 1.3^c^	74.60 ± 1.6^c^	4.78 ± 1.6^c^
PHBV + 15 wt.% Eugenol	160.8 ± 3.3^e^	293.3 ± 1.9^c^	76.36 ± 2.1^c^^,^ ^d^	7.02 ± 2.1^d^

a−e *Different superscripts within the same column indicate significant differences among samples (p < 0.05)*.

In order to better ascertain the thermal protection offered by the electrospun PHBV fibers, the mass loss of the samples was determined at 230°C since, at this temperature, eugenol was completely degraded (mass loss > 98%). This is in agreement with the findings reported by Cao et al. (32), who indicated that neat eugenol completely degrades at 225°C with a weight loss of 96%. Alternatively, it can be observed that PHBV remained highly stable at 230°C (mass loss < 5%) since this temperature is below its T_onset_. One can observe that the mass loss at 230°C increased from 2.15%, for the electrospun PHBV film containing 2.5 wt% of eugenol, up to 7.02%, for the film with 15 wt% of eugenol. However, one can also notice that the thermal stability of eugenol was relatively similar in all the PHBV films since the mass loss at 230°C decreased proportionally to the eugenol content. Therefore, the eugenol encapsulated in the PHBV films showed a significantly higher thermal stability than the free essential oil, though certain amount of eugenol was thermally degraded at temperatures above 200°C. Similar results were obtained by da Silva et al. (72), who observed that eugenol entrapped in cellulose nanostructures and then incorporated in the poly(butylene adipate-*co*-terephthalate) (PBAT) prepared by solvent casting improved the thermal stability of pure eugenol. Likewise, the previous reports of our research group agreed with the thermal properties attain in the present study, showing that the thermal stability of essential oils incorporated into PHAs by electrospinning was improved (59, 67, 73). Therefore, the development of thermally stable systems expands the potential use of electrospinning for the design of active food packaging as an alternative to melt processing technologies such as extrusion or compression molding.

#### Antimicrobial Activity

Table 2 shows the antimicrobial results of the electrospun PHBV monolayers containing different concentrations of eugenol evaluated after 24 h in the open system. This assessment was performed to determine the optimal concentration of eugenol in the PHBV monolayers to, thereafter, develop the multilayer active system. The antimicrobial results of the electrospun PHBV monolayers containing 2.5 and 5 wt.% eugenol showed both significant reduction values (*R* ≥ 1 and < 3), whereas eugenol concentrations of 10 and 15 wt.% showed both a strong reduction (*R* ≥ 3) against *S. aureus*. Similar results were obtained for *E. coli*, in which the reduction was slight (*R* ≥ 0.5 and < 1) for 2.5 wt.% eugenol and significant (*R* ≥ 1 and < 3) for 5, 10, and 15 wt.% eugenol. A high inhibition was obtained for contents of 15 wt.% of eugenol, reaching a reduction of 3.35 and 2.90 against *S. aureus* and *E. coli*, respectively. Differences observed for both bacteria in the antimicrobial performance of eugenol have been ascribed to variances in the action mechanism of essential oils in the bacterial cell membrane and cytoplasm (38, 74). In particular, G- bacteria have higher resistance than G+ ones due to the cell membrane structure is based on complex lipopolysaccharides that restrict the diffusion rate of hydrophobic compounds of essential oils across the cell membranes. Besides, the solubility rate and the concentration of antimicrobial agents in the lipid moiety of the cell membranes, along with the hydrophobicity of the membrane surfaces, can all influence in the resistance of G- bacteria against these active compounds (75–77).

**Table 2:** Antibacterial reduction (R) for *Staphylococcus aureus* (*S. aureus*) and *Escherichia coli* (*E. coli*) of the electrospun monolayers of poly(3-hydroxybutyrate-*co*-3-hydroxyvalerate) (PHBV) containing different amounts of eugenol in the open system for 24 h.

**Microorganism**	**Eugenol** **(wt.%)**	**PHBV monolayer** **Log (CFU/mL)** ***t* = 0 h**	**PHBV monolayer** **Log (CFU/mL)** ***t* = 24 h**	**Active monolayer** **Log (CFU/mL)**	***R***
*S. aureus*	2.5	6.82 ± 0.33	6.80 ± 0.56	5.16 ± 0.12	1.64 ± 0.50
	5			4.21 ± 0.21	2.59 ± 0.32
	10			3.79 ± 0.70	3.01 ± 0.66
	15			3.45 ± 0.32	3.35 ± 0.99
*E. coli*	2.5	6.76 ± 0.51	6.78 ± 0.63	5.79 ± 0.21	0.99 ± 0.78
	5			5.03 ± 0.18	1.75 ± 0.25
	10			4.31 ± 0.43	2.47 ± 0.36
	15			3.88 ± 0.37	2.90 ± 0.81

Based on the antimicrobial reduction attained for both strains and also taken into account the morphological and thermal stability results described above, one can consider that the best concentration of eugenol in the electrospun monolayer of PHBV is 15 wt.%. Hence, the PHBV monolayer films containing 15 wt.% were evaluated against *S. aureus* and *E. coli* strains in an open a closed system for a longer period, that is, up to 15 days. These conditions resemble better those found in actual packaging systems in terms of design and time so that the antimicrobial performance of the active materials can be more accurately determined. As one can observe in Table 3, the electrospun PHBV monolayers presented a strong inhibition (*R* ≥ 3) against *S. aureus* and a significant inhibition (*R* ≥ 1 and < 3) against *E. coli* during the 15 days of evaluation, showing lower antibacterial performance in the open system. The different results obtained for the two tested systems, that is, the open system and the close one (actual packaging) are related with the expected accumulation of the volatile compounds in the headspace of the closed chamber, which can contribute to inhibiting the bacteria growth on the film surfaces. In this regard, Navikaite-Snipaitienea et al. (78) demonstrated that the release of eugenol to the headspace of sealed containers was very rapidly within 1 day and saturation in the packaging headspace was reached after 3 days. The coatings containing the highest amount of eugenol also showed the highest saturation concentration of active substance in the headspace. Similar results were reported in our previous study using oregano essential oil (OEO) and rosemary and green tea natural extracts, in which a slightly higher inhibition was achieved in the closed system in comparison with the open one after 15 days of storage against *S. aureus* and *E. coli* (59). Therefore, the here-attained release and accumulation of volatiles compounds from the developed packaging system can be regarded as an advantage to better preserve food products during their shelf life period.

**Table 3:** Antibacterial reduction (R) for *Staphylococcus aureus* (*S. aureus*) and *Escherichia coli* (*E. coli*) of the electrospun monolayers of poly(3-hydroxybutyrate-*co*-3-hydroxyvalerate) (PHBV) containing 15 wt.% of eugenol in the open and closed systems for 1, 8, and 15 days.

**Microorganism**	**Day**		**Open system**			**Closed system**		
		**PHBV monolayer** **Log (CFU/mL)** ***t* = 0 h**	**PHBV monolayer** **Log (CFU/mL)** ***t* = 24 h**	**Active** **monolayer** **Log (CFU/mL)**	***R***	**PHBV monolayer** **Log (CFU/mL)** ***t* = 24 h**	**Active** **monolayer Log** **(CFU/mL)**	***R***
*S. aureus*	1	6.75 ± 0.04	6.72 ± 0.002	3.49 ± 0.081	3.23 ± 0.05	6.72 ± 0.02	3.49 ± 0.08	3.23 ± 0.12
	8	6.64 ± 0.05	6.62 ± 0.007	3.31 ± 0.033	3.31 ± 0.07	6.70 ± 0.05	3.15 ± 0.07	3.55 ± 0.06
	15	6.77 ± 0.02	6.74 ± 0.010	3.33 ± 0.092	3.41 ± 0.11	6.74 ± 0.06	3.08 ± 0.06	3.66 ± 0.07
*E. coli*	1	6.80 ± 0.09	6.78 ± 0.011	4.08 ± 0.027	2.70 ± 0.07	6.78 ± 0.07	4.08 ± 0.07	2.70 ± 0.05
	8	6.71 ± 0.07	6.69 ± 0.041	3.87 ± 0.084	2.82 ± 0.12	6.75 ± 0.06	3.83 ± 0.09	2.92 ± 0.06
	15	6.77 ± 0.08	6.76 ± 0.030	3.81 ± 0.030	2.95 ± 0.10	6.76 ± 0.08	3.40 ± 0.08	3.36 ± 0.09

### Characterization of the Active Multilayer

#### Morphology

**Figure 6:**
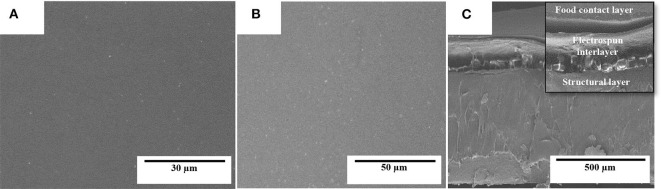
Scanning electron microscopy (SEM) micrographs of the multilayer structure of poly(3-hydroxybutyrate) (PHB) sheet/electrospun poly(3-hydroxybutyrate-*co*-3-hydroxyvalerate) (PHBV) interlayer containing eugenol/PHBV film: **(A)** bottom view of the structural layer of PHB: **(B)** top view of the food contact layer of PHBV; **(C)** multilayer cross-section.

Figure 6 shows the SEM micrographs in the top view and cross-section of the multilayer structures prepared with the electrospun interlayer of PHBV containing 15 wt.% of eugenol. The selected electrospun monolayer was incorporated in a sandwich-type structure between the cast-extruded PHB sheet (structural layer) and the 25-μm PHBV film (food contact layer). One can observe that both the PHB sheet (Figure 6A) and PHBV film (Figure 6B), which constitute the external layers of the multilayer structure, exhibited a smooth and homogenous surface. Figure 6C shows the cross-section of the multilayer system, where the electrospun PHBV monolayer can be seen as an interlayer effectively adhered to both external layers. The high adhesion achieved can be ascribed to the high surface-to-volume ratio of the electrospun fibers, in the submicron range, which efficiently adhered to the contact layer during the thermal post-treatment (annealing). Other recent studies have reported the potential of the electrospinning technology to develop multilayer structures based on the particular morphology of the fibers, even though different materials were employed to produce the layers. For instance, Cherpinski et al. (55) developed multilayer packaging structures based on paper/poly(vinyl alcohol) (PVOH)/PHB. It was observed that the multilayer structures self-adhered to the paper substrate during annealing and the applied electrospun biopolymer coatings yielded a significant improvement of the paper barrier properties to water and limonene vapors. In another study, Fabra et al. (79) studied the effect of different electrospun biopolyester coatings of poly(ε-caprolactone) (PCL), PLA, and PHB on the properties of thermoplastic corn starch (TPCS) films. The multilayers developed showed that the addition of electrospun biopolyester coatings led to an exponential oxygen gas and water vapor permeability reduction as the thickness of the electrospun coating increased. Fabra et al. (53) also obtained multilayer structures based on PHBV prepared by compression-molding and casting, which contained a high barrier interlayer made of electrospun zein nanofibers, concluding that the addition of a zein interlayer significantly improved oxygen barrier properties of the multilayer films prepared by both processing technologies. Similar results were formerly obtained by the use of multilayers of PLA film/electrospun zein fibers/PLA film, in which the introduction of the electrospun zein mat enhanced the barrier properties due to the large aspect ratio of the electrospun fibers by establishing a tortuous pathway for the diffusion of the oxygen gas molecules (51, 52). In another study, Wan et al. (80) developed bacterial cellulose multilayer films by incorporating interlayers of electrospun zein fibers, which enhanced water resistance properties of the multilayer films.

#### Water Contact Angle

**Figure 7:**
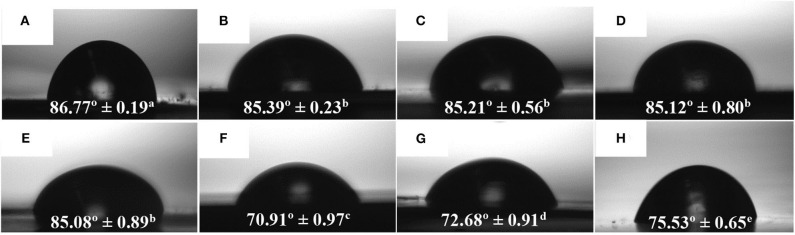
Water contact angle (WCA) of electrospun monolayer films of poly(3-hydroxybutyrate-*co*-3-hydroxyvalerate) (PHBV) containing eugenol: **(A)** Neat PHBV; **(B)** 2.5 wt.%; **(C)** 5 wt.%; **(D)** 10 wt.%; **(E)** 15 wt.%; **(F)** Food contact layer of PHBV, **(G)** Structural layer of poly(3-hydroxybutyrate) (PHB); **(H)** Multilayer containing 15 wt.% eugenol measured on the food contact layer side. ^(*a*−*e*)^ Different superscripts indicate significant differences among samples (*p* < 0.05).

The WCA values of the electrospun PHBV monolayers with different eugenol contents and the multilayer containing 15 wt.% of eugenol are presented in Figure 7. Figure 7A shows the contact angle for the electrospun PHBV monolayer without eugenol, showing a value of 86.77°. A similar WCA value, that is, 86°, was reported by Yoon et al. (81) for PHBV surfaces prepared by the electrospinning technique. As one can observe in Figures 7B–E, the values of WCA of the monolayers containing 2.5 wt.% (85.39°), 5 wt.% (85.21°), 10 wt.% (85.12°), and 15 wt.% of eugenol (85.08°) were very similar and slightly lower than that of PHBV without eugenol. The lower values can be ascribed to the reduction of the surface tension of the water drops on the film surface by the oily molecules of eugenol. It can also be observed that the food contact layer showed an WCA value of 70.91° (Figure 7F), whereas the structural layer of PHB presented a value of 72.68° (Figure 7G). The difference observed in hydrophobicity between the homopolyester and copolyester films are slight, being both hydrophobic polyesters, since the molar content of 3HV in the commercial copolyester is relatively low (82–84). In an application context, Chang et al. (85) studied the WCA of plasma-treated and untreated PHB and PHBV films fabricated by solvent casting. It was observed that the untreated PHB and PHBV films led to WCA values of 76.0 and 72.5°, respectively, and the value decreased in the CH_4_/O_2_ plasma-treated PHB (23.0°) and PHBV (18.5°) films. Finally, the active multilayer containing 15 wt.% eugenol in the electrospun interlayer of PHBV presented a WCA value of 75.53° (Figure 7H). This intermediate value between the external layers of the commercial PHAs and the other electrospun monolayers can be explained in terms of the high eugenol content present in the interlayer, in which some essential oil could diffuse to the external food contact layer of PHBV and decreased its surface tension. In any case, all the samples yielded angles characteristic of hydrophobic materials according to the classification reported by Medina-Jaramillo et al. (86), where ‘hydrophobic’ and ‘hydrophilic’ are defined for angles >65 and < 65°, respectively.

#### Mechanical Adhesion

Table 4 shows the mechanical parameters in terms of tensile modulus (*E*), tensile strength at yield (σ_*y*_), and elongation at break (ε_*b*_) of the electrospun PHBV monolayer films containing eugenol. The table also includes the tensile properties of the cast-extruded sheet of PHBV, which constitutes the structural layer and, at the end, determines the whole mechanical properties of the multilayer. The tensile parameters of the structural layer were similar to those reported earlier for PHB films prepared by extrusion and thermo-compression, that is, ~2,900 MPa, 37 MPa, and 4% (25). Alternatively, the neat PHBV monolayer obtained by electrospinning and subsequent annealing presented E, σ_*y*_, and ε_*b*_ values of 1,252 MPa, 18.1 MPa, and 2%, respectively, which are in the range of the values reported by Corre et al. (87) for melt-extruded films. The incorporation of low contents of eugenol into the electrospun monolayers slightly improved all the mechanical properties, more notably the film elasticity. In particular, the electrospun PHBV monolayer film containing 2.5 wt.% of eugenol presented the highest E value, that is, 2,884 MPa. Lower values were attained, however, at the highest content of eugenol, that is, 15 wt.%, which correlates well with the thermal properties reported above and suggests that the oil particles agglomerated in the biopolymer matrix and induced a plasticizing effect. Furthermore, the differences attained among the electrospun monolayers can also be ascribed to interactions between the coalesced fibers in the electrospun film sample such as slip of fibers over one another, point bonding, alignment, etc. (88). In comparison to PHA films containing essential oils prepared by solvent casting and melting routes, the here-attained electrospun PHBV films were more ductile but less mechanical resistant. For instance, melt-mixed PHB films prepared by Arrieta et al. (89) showed an *E* value of 1,390 MPa, also showing a significant reduction after the incorporation of 15 wt.% of limonene since it is a strong plasticizer that contributes to reducing the intramolecular bonds of the biopolymer. In the work performed by Narayanan et al. (90), solvent-casted PHB films with different amounts of eugenol showed a σ_*y*_ reduction from 40 MPa, for neat PHB, to 23.5 MPa after the incorporation of the essential oil. In another study, compression-molded bilayer structures based on PHBV containing different essential oils at 15 wt% were produced by Requena et al. (91), showing that the E and σ_*y*_ values ranged between 1,141–773 MPa and 27.6–17 MPa, respectively. These results confirm that the electrospun film show lower mechanical strength due to they consist of coalesced fibers and also show higher porosity (92). In any case, all the developed monolayers showed sufficient strength to be successfully applied as interlayers in packaging applications.

**Table 4:** Mechanical properties of the cast-extruded poly(3-hydroxybutyrate) (PHB) sheet and electrospun monolayer films of poly(3-hydroxybutyrate-*co*-3-hydroxyvalerate) (PHBV) containing eugenol in terms of tensile modulus (*E*), tensile strength at yield (**σ**_*y*_), and elongation at break (**ε**_*b*_).

**Sample**	***E* (MPa)**	****σ**_y_(MPa)**	****ε**_b_(%)**
Structural PHB layer	3014 ± 86^a^	29.1 ± 3.3^a^	1.4 ± 0.3^a^
PHBV monolayer	1252 ± 79^b^	18.1 ± 2.1^b^	2.0 ± 0.2^b^
PHBV + 2.5 wt.% eugenol monolayer	2884 ± 70^c^	20.6 ± 4.8^c^	2.1 ± 0.2^b^
PHBV + 5 wt.% eugenol monolayer	2632 ± 73^d^	21.3 ± 0.8^c^	2.2 ± 0.1^b^^,^ ^c^
PHBV + 10 wt.% eugenol monolayer	2261 ± 29^e^	28.3 ± 1.4^d^	2.4 ± 0.7^c^^,^ ^d^
PHBV + 15 wt.% eugenol monolayer	1897 ± 51^f^	26.5 ± 1.8^c^^,^ ^d^	2.5 ± 0.4^d^

a−f *Different superscripts within the same column indicate significant differences among samples (p < 0.05)*.

Furthermore, the adhesion capacity of the electrospun PHBV layer to the structural and food contact layers was assessed by T-peel adhesion tests. For all the samples studied, the PHB film broke before the detachment of the adhesive union, thus revealing an excellent performance of the electrospun PHBV interlayers as tie-layers. A maximum stress of ~10 N/cm was reached at break of the PHB film, thus proving an adhesion force above that value.

#### Barrier Performance

The WVP and LP values of the electrospun PHBV monolayers containing eugenol, food contact PHBV film, and structural PHB sheet are shown in Table 5. In the case of the food contact film, the permeability values were 6.27 × 10^−14^ and 5.17 × 10^−14^ Kg· m·m^−2^·s^−1^·Pa^−1^ for water and limonene vapors, respectively. These values were higher than those observed for both the electrospun PHBV monolayer without eugenol, that is, 4.05 × 10^−14^ and 3.75 × 10^−14^ Kg·m·m^−2^·s^−1^·Pa^−1^, respectively, and also for the structural layer of PHB, that is, 1.75 × 10^−15^ and 1.95 × 10^−15^ Kg·m·m^−2^·s^−1^·Pa^−1^. This observation is related to the higher 3HV molar content of the biopolyester in the food contact layer, that is, 8 mol.%. In a context of packaging applications for food preservation, the present PHAs showed values of permeability relatively close to petroleum derived thermoplastics such as polyethylene terephthalate (PET), with WVP value around 5.2 × 10^−15^ Kg·m·m^−2^·s^−1^·Pa^−1^ (54, 87). As described during the mechanical analysis, the differences attained can be ascribed to the remaining porosity and, thus, lower continuity of the annealed fiber-based material (92). One can also observe that the incorporation of eugenol into the PHBV monolayers decreased the permeability of both vapors whereas there were also significant differences for the electrospun monolayers containing eugenol. The monolayer PHBV films containing 2.5 and 5 wt.% of eugenol presented WPV values of 3.59 × 10^−14^ and 2.79 × 10^−14^ Kg·m·m^−2^·s^−1^·Pa^−1^ and LP values of 2.86 × 10^−14^ and 2.13 × 10^−14^ Kg·m·m^−2^·s^−1^·Pa^−1^. For the monolayer PHBV films containing 10 wt.% and 15 wt.% of eugenol, the WVP values decreased to 1.98 × 10^−14^ and 0.95 × 10^−14^ Kg·m·m^−2^·s^−1^·Pa^−1^, respectively. In the case of LP, values were respectively 1.83 × 10^−14^ and 0.81 × 10^−14^ Kg·m·m^−2^·s^−1^·Pa^−1^. Since water vapor is mainly a diffusivity-driven property in PHAs due to their low water sorption nature (93), the decrease in permeability to water vapor can be ascribed to the hydrophobic character of eugenol, where the presence of the oily molecules dispersed in PHA matrix impaired the mobility of water molecules (61, 67, 94). For limonene, as opposed to moisture, the aroma molecules are known to plasticize PHAs and, then, solubility plays a more important role in permeability than diffusion. Therefore, the lower permeability values attained indicate that eugenol also reduced the sorption of limonene in the PHBV matrix. Similar barrier improvements were reported by Melendez-Rodriguez et al. (67) when incorporated eugenol encapsulated in mesoporous silica nanoparticles in PHBV monolayers, though the barrier effect was mainly ascribed to the mineral nanofillers. Likewise, Hasheminya et al. (95) showed that Kefiran/carboxymethyl cellulose composite films incorporating *Satureja Khuzestanica* essential oil by solvent casting decreased significantly the WVP values due to interactions between the hydrophobic compounds and the biopolymer. This phenomenon was explained by a reduction of the number of hydrophilic groups available that could diffuse into the continuous biopolymer phase.

**Table 5:** Permeance of water and limonene vapors and limonene of the structural poly(3-hydroxybutyrate) (PHB) layer, food-contact poly(3-hydroxybutyrate-*co*-3-hydroxyvalerate) (PHBV) layer, electrospun PHBV interlayers with different contents of eugenol, and multilayer with 15 wt.% eugenol.

**Sample**	**Thickness (μm)**	**Permeability**		**Permeance**	
		**Water vapor × 10^**14**^** **(Kg·m·m^−2^·s^−1^·Pa^−1^)**	**Limonene vapor × 10^**14**^** **(Kg·m·m^−2^·s^−1^·Pa^−1^)**	**Water vapor × 10^**10**^** **(Kg·m^−2^·s^−1^·Pa^−1^)**	**Limonene vapor × 10^**10**^** **(Kg·m^−2^·s^−1^·Pa^−1^)**
Food contact PHBV layer	25 ± 3	6.27 ± 0.15^a^	5.17 ± 0.65^a^	25.08 ± 0.59^a^	20.68 ± 0.26^a^
Structural PHB layer^*^	500 ± 7	0.18 ± 0.50^b^	0.20 ± 0.10^b^	0.04 ± 0.01^b^	0.04 ± 0.01^b^
PHBV monolayer	70 ± 1	4.05 ± 0.13^c^	3.75 ± 0.93^c^	5.87 ± 0.19^c^	5.44 ± 0.13^c^
PHBV + 2.5 wt.% eugenol monolayer	71 ± 1	3.59 ± 0.54^d^	2.86 ± 0.42^d^	5.06 ± 0.77^c^	4.03 ± 0.59^d^
PHBV + 5 wt.% eugenol monolayer	73 ± 1	2.79 ± 0.46^e^	2.13 ± 0.14^e^	3.83 ± 0.63^d^	2.92 ± 0.19^e^
PHBV + 10 wt.% eugenol monolayer	72 ± 3	1.98 ± 0.37^f^	1.83 ± 0.11^e^	2.74 ± 0.51^e^	2.54 ± 0.15^e^
PHBV + 15 wt.% eugenol monolayer	71 ± 1	0.95 ± 0.13^g^	0.81 ± 0.48^f^	1.33 ± 0.18^f^	1.14 ± 0.13^f^
Multilayer	580 ± 12	—	—	0.03 ± 0.01^g^	0.02 ± 0.10^g^

a−g *Different superscripts within the same column indicate significant differences among samples (p < 0.05)*.

* *Permeability values reported elsewhere (27)*.

The permeance of all the monolayers and multilayer was also determined by dividing the permeability values by each sample thickness. Permeance is the time it takes the vapor to transmit through a unit area of film that has a vapor pressure difference between the two exposed surfaces of the film, therefore, the lower the permeance the higher the barrier of the film or sheet. One can observe that the water vapor and limonene permeances of the active multilayer were 0.03 × 10^−10^ and 0.02 × 10^−10^ Kg·m^−2^·s^−1^·Pa^−1^, respectively. These values were slightly lower than those attained for the structural PHB sheet due to the presence of electrospun interlayers of PHBV containing eugenol and the higher thickness of the sample. The present multilayers can be compared with the multilayers prepared by Akinalan Balik et al. (58), who evaluated the water permeance of multilayer films that consisted of an electrospun pectin-based interlayer sandwiched between two electrospun PHBV films. The previous study reported higher permeance values for the neat PHBV/PHBV film (5 × 10^−10^ Kg·m^−2^·s^−1^·Pa^−1^) mainly due to the lower total thickness of the multilayer (72 μm). In the case of PHBV/electrospun pectin/PHBV multilayer, the permeance value was also lower (1.75 × 10^−10^ kg·m^−2^·Pa^−1^·s^−1^) even though the electrospun pectin highly improved the barrier performance of PHBV. Therefore, the here-prepared multilayers can be applied to medium barrier applications or even medium-to-high barrier applications at the current thickness.

#### Antimicrobial Activity

Table 6 shows the antimicrobial properties of the multilayer system against *S. aureus* and *E. coli* strains in an open a closed system for up to 15 days. It can be observed that the multilayer presented a significant inhibition reduction (*R* ≥ 1 and < 3) against both bacterial strains in the all days of evaluation, reaching at day 15 *R* values of 1.84 for *S. aureus* and 1.47 for *E. coli*. Compared with the monolayer materials, lower values were obtained since the release rate was slower due to the presence of the food contact layer, which potentially hindered the diffusion of eugenol (96). In any case, the *R* values obtained for these multilayers systems, ranging between 1.24 and 2.19, were still significant (*R* ≥ 1 and < 3) against both bacteria and in both tested systems. Similarly, Cerqueira et al. (61) developed active multilayers based on bilayers of PHBV/zein and PHBV containing cinnamaldehyde by electrospinning, achieving a good antimicrobial activity against *L. monocytogenes* with a logarithmic reduction around of 2.18 Log_10_ CFU/mL after 30 days of storage. Figueroa-Lopez et al. (42) also achieved a high reduction of *S. aureus* [3.9 Log_10_ (CFU/mL)] using active multilayer films based on gelatin, PCL, and black pepper oleoresin prepared by electrospinning. It is also worthy to note that the inhibition had a slight increase in the closed system at days 8 and 15 of evaluation, which is related to the accumulation of eugenol released from the multilayer in the headspace. Therefore, development of active packaging are foreseen to preserve food products for up to 15 days, maintaining their physical and microbiological quality (97). Thus, the design of multilayer active systems can successfully allow the controlled release of the bioactive compounds.

**Table 6:** Antibacterial activity for *S. aureus* and *E. coli* of the multilayer structure of poly(3-hydroxybutyrate) (PHB) sheet/electrospun poly(3-hydroxybutyrate-*co*-3-hydroxyvalerate) (PHBV) interlayer containing eugenol/PHBV film in the open and closed systems for 15 days.

**Microorganism**	**Day**		**Open system**			**Closed system**		
		**Control** **PHBV** **Log (CFU/mL)** ***t* = 0 h**	**Control** **PHBV** **Log (CFU/mL)** ***t* = 24 h**	**Active multilayer** **Log (CFU/mL)**	***R***	**Control** **PHBV** **Log (CFU/mL)** ***t* = 24 h**	**Active multilayer** **Log (CFU/mL)**	***R***
*S. aureus*	1	6.75 ± 0.04	6.72 ± 0.002	5.21 ± 0.05	1.51 ± 0.08	6.72 ± 0.02	5.21 ± 0.05	1.51 ± 0.08
	8	6.64 ± 0.05	6.62 ± 0.007	5.01 ± 0.05	1.61 ± 0.13	6.70 ± 0.05	4.76± 0.07	1.94 ± 0.10
	15	6.77 ± 0.02	6.74 ± 0.010	4.90 ± 0.02	1.84 ± 0.07	6.74 ± 0.06	4.55 ± 0.02	2.19 ± 0.13
*E. coli*	1	6.80 ± 0.09	6.78 ± 0.011	5.54 ± 0.06	1.24 ± 0.06	6.78 ± 0.07	5.54 ± 0.06	1.24 ± 0.09
	8	6.71 ± 0.07	6.69 ± 0.041	5.41 ± 0.07	1.28 ± 0.13	6.75 ± 0.06	5.26 ± 0.08	1.49 ± 0.11
	15	6.77 ± 0.08	6.76 ± 0.030	5.29 ± 0.09	1.47 ± 0.17	6.76 ± 0.08	5.05 ± 0.04	1.71 ± 0.07

In a food packaging content, it is also worthy to mention that a slow or sustained release supposes an advantage because, in some cases, the excess of volatiles substances can be affect the organoleptic food properties and result in consumer rejection. As reported by Ribes et al. (98), the presence of active compounds such as eugenol, carvacrol, and vanillin altered the sensory acceptance of fruit juices. It was observed that active compounds immobilized in silica supports induced a change on the aroma of juice samples, particularly for the samples containing eugenol. Thus, the use of multilayers can also successfully contribute to reducing the organoleptic impact of essential oils on the original aroma of food products.

## Conclusion

A novel multilayer structure fully based on PHA with antibacterial properties was successfully produced by the electrospinning of PHBV fibers containing eugenol on a cast-extruded PHB sheet (structural layer) followed by the deposition of a PHBV film (food contact layer) and thereafter the application of annealing to the whole assembly at mild temperature. The amount of eugenol in the electrospun monolayer was optimal at 15 wt.% since it yielded high electrospinnability and provided the highest antibacterial properties against bacteria strains of *S. aureus* and *E. coli* as well as sufficient thermal stability. The resultant multilayer showed high hydrophobicity to be used in high humidity packaging environments, sufficient interlayer adhesion, a mechanical performance similar to the structural layer, and improved barrier properties against water and aroma vapors. The antimicrobial tests finally showed that the multilayer is very effective to reduce and control the growth of food-borne bacteria in both open and closed systems for up to 15 days. Moreover, through the multilayer design, the release of eugenol was sustained and it can be prolonged so that the organoleptic properties would be less affected. Further studies will be focused on analyzing the migration of eugenol from the multilayer active system into food simulants and the practical application of the multilayer in the form of packaging articles such as trays or lids to study the shelf life of food products and also their impact on the organoleptic properties.

## Data Availability Statement

The raw data supporting the conclusions of this article will be made available by the authors, without undue reservation.

## Author Contributions

KF-L performed all experiments, measurements, data analysis, and wrote the manuscript. LC realized the mechanical test of the materials. JL and ST-G proposed, planned, guided the execution of the research work, and co-wrote the manuscript. All authors contributed to the article and approved the submitted version.

## Conflict of Interest

The authors declare that the research was conducted in the absence of any commercial or financial relationships that could be construed as a potential conflict of interest.
